# Infecção por COVID-19 em Gestante Cardiopata

**DOI:** 10.36660/abc.20200517

**Published:** 2020-11-01

**Authors:** Lucianna Serfaty de Holanda, Laíses Vieira, Maria Talita Campos, Vitor Bruno Teixeira de Holanda, Ivy Almeida Cavalcante e Silva, Daniel Serfaty, Feliciano Mendes Vieira

**Affiliations:** 1 Fundação Hospital de Clínicas Gaspar Vianna do Pará BelémPA Brasil Fundação Hospital de Clínicas Gaspar Vianna do Pará, Belém, PA - Brasil; 2 Centro Universitário do Estado do Pará BelémPA Brasil Centro Universitário do Estado do Pará, Belém, PA - Brasil; 3 Hospital do Coração do Pará BelémPA Brasil Hospital do Coração do Pará, Belém, PA - Brasil; 4 Hospital Metropolitano de Urgência e Emergência do Pará BelémPA Brasil Hospital Metropolitano de Urgência e Emergência do Pará, Belém, PA – Brasil

**Keywords:** Gravidez de Alto Risco, Cardiopatias/complicações, Insuficiência Respiratória, COVID-19, Coronavírus, Síndrome Respiratória Aguda Grave

## Introdução

Sobre os aspectos obstétricos da infecção COVID-19, é necessário considerar que ela é uma doença recente, não havendo conhecimento específico acerca do assunto para a elaboração de protocolos assistenciais. Em decorrência disso, várias orientações baseiam-se na comparação com infecções causadas por outros vírus (SARS-CoV, MERS-CoV e H1N1); portanto, tudo o que existe de evidências atualmente está sujeito a modificações a partir de novas descobertas.

As infecções causadas por SARS-CoV e MERS-CoV foram limitadas regionalmente, mas os poucos casos obstétricos publicados apontam a necessidade de suporte avançado de vida para as gestantes com grave comprometimento do prognóstico materno. Todos realçam a importância dos cuidados com a dispersão do vírus.^1^^–^^4^

Em uma revisão publicada, foram selecionados 23 estudos, somando 32 gestantes e 30 recém-nascidos. As gestantes se apresentaram assintomáticas em 22% dos casos, mas 6% necessitaram de cuidados de suporte avançado de vida em unidade de terapia intensiva (UTI). A via de parto foi a cesárea em 27 mulheres, e 47% dos partos ocorreram antes de 36 semanas de gestação. Os autores informaram que nenhum caso de morte materna foi observado nessa revisão. Além disso, outra extensa revisão apontou que até o momento não foi confirmado nenhum caso de transmissão vertical desse vírus.^5^

Há relatos limitados de séries de casos sobre o impacto dos Coronavírus durante a gravidez. Nas mulheres afetadas por síndrome respiratória aguda grave (SRAG) ou síndrome respiratória do Oriente Médio (SROM), a taxa de mortalidade apareceu mais alta nas que foram afetadas durante a gestação, em comparação com as não grávidas.^6^

## Descrição

Paciente gestante, 26 anos, G3P1A1, com antecedente de valvopatia mitral reumática com troca de valva mitral biológica há 9 anos. Histórico de internações anteriores, em 2014, para realização de parto pré-termo cesariana. Naquele momento, a paciente encontrava-se assintomática, do ponto de vista cardiovascular; por isso, não houve indicação de contracepção definitiva por parte da equipe que a avaliou.

Depois disso, a paciente seguiu em acompanhamento ambulatorial na cardiologia. Em uso de Carvedilol, 3,125 mg 12/12h; AAS, 100 mg/dia; e Penicilina G benzatina, 1.200.000 UI a cada 21 dias.

Em 31 de outubro de 2019, a mulher apresentou necessidade de internação hospitalar, dando entrada no serviço de pronto-atendimento cardiológico devido a novo quadro gestacional de 16 semanas, evoluindo com taquicardia ventricular sustentada com frequência cardíaca de 140 batimentos por minuto (bpm), pressão arterial de 120 x 70 mmHg, sem sinais clínicos de instabilidade hemodinâmica.

O resultado dos exames laboratoriais do dia da internação estava sem sinais de anemia, distúrbios da coagulação, infecção, distúrbios hidroeletrolíticos e disfunção hepática ou renal. Em ultrassonografia (USG) obstétrica de feto único, não havia sinais de sofrimento fetal.

Após compensação da arritmia e do quadro clínico, a gestante permaneceu internada, foi acompanhada pela equipe de obstetrícia e avaliada pela cardiologia. Ela realizou um ecocardiograma que evidenciou átrio esquerdo de tamanho aumentado (50 mm), fração de ejeção de 64%, área valvar da prótese de 1,0 cm^2^, gradiente ventrículo esquerdo-átrio esquerdo (VE-AE) 12 e pressão sistólica de artéria pulmonar 40. A paciente recebeu alta médica no dia 5 de novembro de 2019, com orientação de seguimento em ambulatório de pré-natal de alto risco do serviço.

Não obstante, no dia 9 de março de 2020, ela deu entrada na emergência do serviço, apresentando quadro de taquicardia com frequência de 200 bpm e dor em baixo-ventre. O eletrocardiograma evidenciou taquicardia paroxística supraventricular.

Naquela data, o exame físico da gestante, de 33 semanas e 5 dias, estava sem alteração na ausculta pulmonar, sem sinais de instabilidade hemodinâmica, com eupneia em ar ambiente, altura uterina de 33 cm, batimento cardíaco fetal de 129 bpm, colo uterino fechado, posterior e grosso, sem perdas ginecológicas ou sinais de sangramento. Após medidas clínicas, ela retornou ao ritmo sinusal e foi encaminhada à enfermaria obstétrica, onde permaneceu em seguimento com a obstetrícia, sendo avaliada pela cardiologia. Esta indicou escolha do momento de parto a critério da obstetrícia e via de parto cesariana com laqueadura depois, devido ao risco cardiovascular da paciente. Além disso, indicou digitálico em caso de novo evento de taquiarritmia. Assim, a gestante recebeu alta médica no dia 23 de março 2020, com prescrição de carvedilol, 3,125 mg 12/12 h e AAS, 100 mg/dia, bem como manutenção da Penicilina G benzatina a cada 21 dias.

Em 30 de março de 2020, a mulher, agora com 36 semanas e 5 dias de gestação, deu entrada no serviço de emergência cardiovascular da Fundação Hospital de Clínicas Gaspar Vianna (FHCGV), com quadro de febre, tosse produtiva e insuficiência respiratória aguda. O exame físico evidenciou taquipneia em ar ambiente, pressão arterial de 100 x 70 mmHg e ausculta pulmonar com murmúrio vesicular audível com roncos difusos.

O laboratório admissional mostrou as seguintes taxas: hemoglobina, 12,3; hematócrito, 31,6%; leucócitos, 20.900, com segmentados, 87,9%; linfócitos, 5%, sem desvio à esquerda; plaquetas, 137.000; índice internacional normalizado (INR), 1; tempo de protrombina, 11,7 segundos; tempo de tromboplastina parcial ativada (TTPA), 35 segundos; gasometria arterial pós-intubação orotraqueal com pH 7,26; pressão parcial de gás carbônico sanguíneo (PCO_2_), 50 mmHg; pressão parcial de oxigênio sanguíneo (PO_2_), 136 mmHg; bicarbonato sanguíneo (HOC3), 22 mEq/L; Excesso de base, −4; saturação de oxigênio sanguíneo (SatO_2_), 98%; lactato, 1,2 mmol/l; creatinina, 0,7 g/dl; sódio, 135 mmol/l; potássio, 3,5; magnésio, 1,8; cálcio iônico, 1,13; bilirrubinas totais, 1,36; bilirrubina direta, 0,85; bilirrubina indireta, 0,51; transaminase glutâmico-oxalacética (TGO), 27; transaminase glutâmico-pirúvica (TGP), 20; reação em cadeia da polimerase (PCR), 5 (VR < 5).

**Figura 1:**
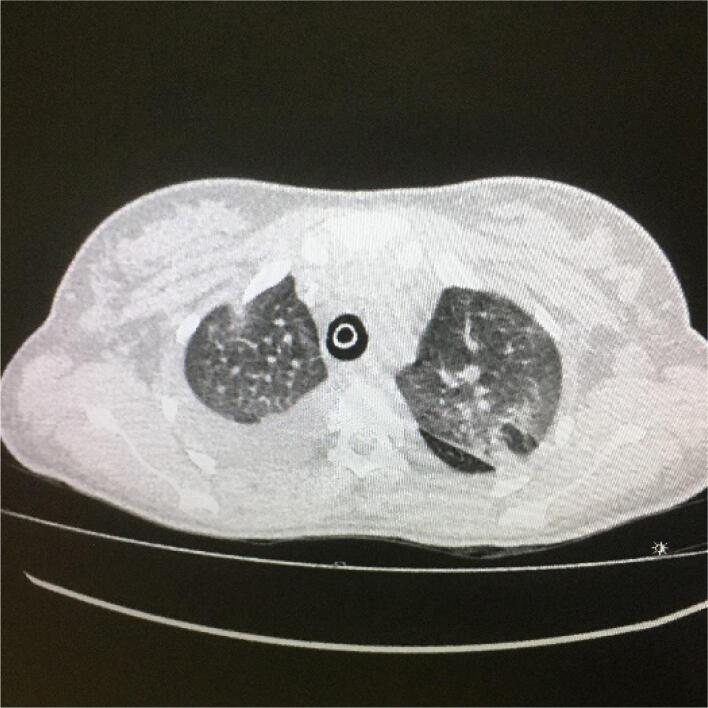
Tomografia de tórax da paciente, porção do ápice com evidência de vidro fosco difuso, predomínio periférico e derrame pleural bilateral.

**Figura 2:**
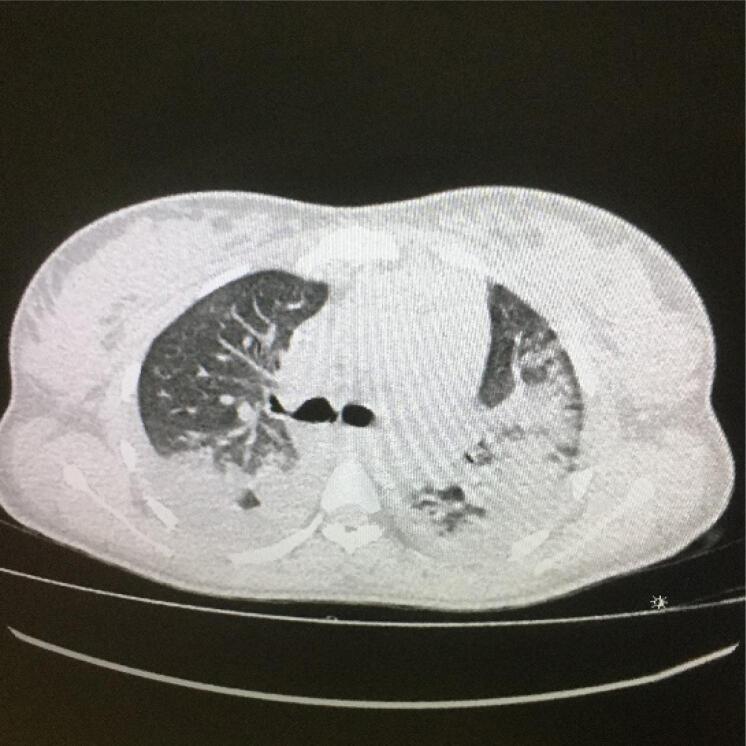
Tomografia de tórax da paciente, 1/3 médio com evidência de vidro fosco difuso, também com periférico e derrame pleural bilateral.

Tomografia de tórax da paciente, com porção do ápice com evidência de vidro fosco difuso, predomínio periférico e derrame pleural bilateral (Figura 1), além de 1/3 médio com evidência de vidro fosco difuso, também periférico e derrame pleural bilateral (Figura 2).

Foi realizada intubação orotraqueal, colocada em pressão controlada, pressão expiratória final positiva (PEEP) 9, frequência respiratória de 20 irpm, volume corrente 300, fração inspirada de oxigênio (FiO) 30%, com uma relação PO_2_/FiO_2_ de 388. Também se coletou **swab** nasofaríngeo e oral, e foi feito PCR para COVID-19 e H1N1. Posteriormente, a paciente foi encaminhada para cesariana e laqueadura de urgência. A cirurgia foi feita sob anestesia geral.

Depois disso, a mulher foi encaminhada à UTI, permanecendo sedada e com escala de RASS −5. Ela evoluiu com instabilidade hemodinâmica e tempo de enchimento capilar de mais de 2 segundos, requerendo a introdução de substâncias vasoativas (norepinefrina e dobutamina). Foi iniciada, então, prescrição de olsetamivir, 75 mg 12/12 h, azitromicina, 500 mg/dia, hidroxicloroquina, 400 mg 12/12 h, piperacilina-tazobactam, 4,5 g 6/6 h, furosemida, 2 ampolas 8/8 h, carbegolina dose única para inibir lactação e profilaxia para tromboembolismo pulmonar (TEP) com enoxaparina, 40 mg/dia.

Após o parto, foi realizada tomografia de tórax, que evidenciou presença de consolidação, broncograma aéreo e padrão em vidro fosco em lobos superiores bilaterais, com derrame pleural discreto bilateralmente.

No dia 1º de abril de 2020, a paciente teve melhora clínica, estabilidade hemodinâmica, com possibilidade de suspensão dos fármacos vasoativos, ainda sob ventilação mecânica, mas já em pressão de suporte, apresentando aumento da relação PO_2_/FiO_2_ de 644. A sedação foi pausada pela manhã.

Em 2 de abril de 2020, a paciente foi extubada e colocada em cateter de oxigênio com baixo fluxo. O laboratório do dia mostrou: hemoglobina, 8,4; hematócrito, 255; leucócitos, 10.190; segmentados, 68%; plaquetas, 137.000; gasometria arterial, 7,43; PCO_2_, 35; PO_2_, 119; base excess, 1; bicarbonato, 24; SatO_2_, 99%; lactato, 1,0.

No mesmo dia, o exame de PCR para COVID-19 deu positivo. Em 2 de abril de 2020, a paciente foi transferida para a Fundação Santa Casa de Misericórdia do Pará, serviço referenciado para manejo de pacientes com COVID-19. Lá, deu-se seguimento ao tratamento com olsetamivir, 150 mg/dia, difosfato de cloroquina, 450 mg/dia, azitromicina, 500 mg/dia, e piperacilina-tazobactam, 18 g/dia. No dia 4 de abril de 2020, ela evoluiu com taquiarritmia do tipo supraventricular; no dia 7 entrou em insuficiência respiratória aguda, necessitando novamente de ventilação mecânica invasiva, além de sedação e curarização.

A paciente foi colocada em pronação no dia 9 de abril de 2020, por 16 horas, mas manteve assincronia ventilatória e baixa relação PO_2_/FiO_2_, mesmo após altos títulos de PEEP e FiO_2_. No dia 11 de abril de 2020, ela manteve assincronia ventilatória, SatO_2_ de 50% e baixa relação PO_2_/FiO_2_. Então, evoluiu para parada cardiorrespiratória em assistolia, sem sucesso ante manobras de reanimação, indo a óbito às 10h10.

O recém-nascido nasceu em sofrimento fetal e bradicárdico, necessitando de manobras de reanimação; Apgar 2/6/7, líquido amniótico claro e sem grumos, peso ao nascer 2,750 kg, estatura 50 cm, perímetro cefálico, torácico e abdominal de 34, 32 e 31 cm, respectivamente. Ele foi encaminhado para UTI neonatal ao nascer e permaneceu em ventilação mecânica do dia 30 de março até o dia 8 de abril de 2020. Continuou internado até o dia 21 de abril de 2020, recebendo alta médica restabelecido. Foi feito exame para COVID-19 no RN no dia 21 de abril de 2020, após confirmação do diagnóstico materno por PCR, mas o resultado foi negativo.

## Conclusões

Existem poucos estudos sobre o impacto do novo Coronavírus na gestação, uma vez que as pandemias de SARS e MERS que ocorreram antes foram limitadas geograficamente. Esse foi o primeiro caso de gestante com SARS COVID-19 no norte do estado do Pará, fato esse agravado pela cardiopatia gestacional associada ao quadro infeccioso.

Diante da escassez de dados sobre a infecção por COVID-19 em gestantes e, em especial, gestantes cardiopatas, torna-se imprescindível o estudo e o conhecimento de como essa doença se comporta nesse grupo de pacientes e quais as possíveis consequências tanto para a mãe como para o recém-nascido.
