# Supplementary material for: Infecção por COVID-19 em Gestante Cardiopata
Source: Arq Bras Cardiol. 2020 Nov 1;115(5):936–8. [Article in Portuguese] doi: 10.36660/abc.20200517 (PMC8452208; doi:10.36660/abc.20200517)
Supplement: Translation

# Translation: COVID-19 Infection in a Cardiopatic Pregnant Woman

**Authors:** Lucianna Serfaty de Holanda, Laíses Vieira, Maria Talita Campos, Vitor Bruno Teixeira de Holanda, Ivy Almeida Cavalcante e Silva, Daniel Serfaty, Feliciano Mendes Vieira

**Affiliations:** 1 BelémPA Brazil Fundação Hospital de Clínicas Gaspar Vianna do Pará, Belém, PA - Brazil; 2 BelémPA Brazil Centro Universitário do Estado do Pará, Belém, PA - Brazil; 3 BelémPA Brasil Hospital do Coração do Pará, Belém, PA - Brasil; 4 BelémPA Brazil Hospital Metropolitano de Urgência e Emergência do Pará, Belém, PA – Brazil

**Keywords:** Pregnancy, High-Risk, Heart Diseases/complications, Respiratory Insufficiency, COVID-19, Coronavirus, Severe Acute Respiratory Syndrome

## Introduction

Regarding the obstetric aspects of COVID-19 infection, one should consider that it is a recent disease, with no specific knowledge on the theme to draft medical care protocols. For this reason, various guidelines are based on its comparison with infections caused by other viruses (SARS-CoV, MERS-CoV, and H1N1); therefore, all information concerning current evidence on the issue is subject to change due to new discoveries.

Infections caused by SARS-CoV and MERS-CoV have tended to be regionally bound, but the few obstetric cases published in the literature point to the need for advanced medical support for pregnant women with severe involvements identified in maternal prognoses. All of these highlight the importance of care against the dissemination of the virus.^1^^–^^4^

In a recently published literature review, 23 studies were selected, including 32 pregnant women and 30 newborns. The pregnant women were asymptomatic in 22% of the cases, but 6% required advanced life support medical care in an Intensive Care Unit (ICU). Births were performed through Caesarean section (C-section) in 27 women, and 47% of the births occurred before 36 weeks of gestation. The authors informed that no case of maternal death was observed in this review. Likewise, another extensive literature review pointed out that, to date, no case of the vertical transmission of this virus has been confirmed.^5^

Case series on the impact of Coronavirus during pregnancy are scarce in the literature. In women affected by severe acute respiratory syndrome (SARS) or Middle East respiratory syndrome (MERS), the mortality rate proved to be higher in those affected during pregnancy, as compared to those women who were not pregnant.^6^

## Description

This study examines a 26-year-old, pregnant woman, G3P1A1, with a medical history of rheumatic mitral valve disease, who underwent a biological mitral valve replacement nine years ago. This patient presented a medical history of previous hospitalizations, in 2014, for a C-section, pre-term birth. At that moment, the patient was asymptomatic, from the cardiovascular point of view, and for this reason, there was no recommendation of a definitive contraception prescribed by the team that evaluated the patient.

After, the patient underwent an outpatient cardiologic follow-up, prescribed with Carvedilol, 3,125 mg 12/12h; AAS, 100 mg/day; and Benzathine Penicillin G, 1.200.000 UI, every 21 days.

On October 31, 2019, the woman required hospitalization, and received medical care at the cardiologic emergency clinic due to a new 16-week gestational condition, evolving to sustained ventricular tachycardia, with a heart rate of 140 beats per minute (BPM), blood pressure of 120 x 70 mmHg, with no clinical signs of hemodynamic instability.

The result of the laboratory exams on the day of hospitalization showed no signs of anemia, coagulation disorders, infection, hydro-electrolyte imbalance, or hepatic or renal dysfunction. In an obstetric ultrasound (US) of a single fetus, no signs of fetal distress were observed.

After compensating for both the arrhythmia and the clinical condition, the pregnant woman remained in the hospital, receiving medical care from the obstetrics team and evaluation from the cardiology team. She was submitted to an echocardiogram, which showed evidence of a swollen left atrium (50mm), an ejection fraction of 64%, a prosthesis valve area of 1.0 cm^2^, a left ventricle-left atrium (LV-LA) gradient of 12 and a pulmonary artery systolic blood pressure of 40. The patient received hospital discharge on November 5, 2019, with the medical advice to continue in outpatient follow-up for high-risk pre-natal care.

Nonetheless, on November 9, 2020, the patient was once again admitted to the emergency clinic, with a medical condition of tachycardia, with a heart rate of 200 BPM and lower abdominal pain. The electrocardiogram revealed paroxysmal supraventricular tachycardia.

On that date, the patient's physical exam, of 33 weeks and 5 days, showed no change in pulmonary auscultation, with no signs of hemodynamic instability, with eupnea in ambient air, a uterine height of 33 cm, a fetal heartbeat of 129 BPM, a closed posterior and thick cervix, with no gynecological losses or signs of bleeding. After the clinical measures, she returned to the sinus rhythm and was referred to the obstetric infirmary, where she remained in follow-up with the obstetrics team and was evaluated by the cardiology team. The cardiology team recommended choosing the birth time according to the criteria established by the obstetrics team and through a C-section, with subsequent sterilization due to the patient's cardiovascular risk. This team also recommended the use of cardiac glycosides should a new event of tachycardia occur. Thus, the patient received hospital discharge on March 23, 2020, with a prescription of carvedilol, 3.125 mg 12/12 h and AAS, 100 mg/day, as well as the continuance of Benzathine Penicillin G, every 21 days.

On March 30, 2020, the patient, now at 36 weeks and 5 days of gestation, was admitted to the cardiovascular emergency clinic at Fundação Hospital de Clínicas Gaspar Vianna (FHCGV), with a fever, a productive cough, and severe respiratory insufficiency. The physical exam showed evidence of tachypnea in ambient air, with a blood pressure of 100 x 70 mmHg, and pulmonary auscultation with an audible vesicular murmur with diffuse rumbles.

The admissions laboratory reported the following rates: hemoglobin, 12.3; hematocrit, 31.6%; leukocytes, 20,900, segmented, 87.9%; lymphocytes, 5%; with no left deviation; platelets, 137,000; normalized international ratio (INR), 1; prothrombin time, 11.7 seconds; partial thromboplastin time (PTT), 35 seconds; post-orotreacheal intubation arterial-blood gas, with a pH of 7.26; partial pressure of blood carbon dioxide (PCO_2_), 50 mmHg; partial pressure of blood oxygen (PO_2_), 136 mmHg; blood bicarbonate (HOC3), 22 mEq/L; base excess, −4; saturation of blood oxygen (SatO_2_), 98%; lactate, 1.2 mmol/l; creatinine, 0.7 g/dl; sodium, 135 mmol/l; patassium, 3.5; magnesium, 1.8; ionized calcium, 1.13; total bilirubin, 1.36; direct bilirubin, 0.85; indirect bilirubin, 0.51; aspartate transaminase (AST), 27; glutamic-pyruvic transaminase (GPT), 20; polymerase chain reaction (PCR), 5 (VR < 5).

Tomography of the patient's chest, with apex portion with evidence of diffuse ground glass, peripheral predominance and bilateral pleural effusion (figure 1), in addition to 1/3 medium with evidence of diffuse ground glass, also peripheral and bilateral pleural effusion (figure 2).

**Figure 1:** Patient's chest X-ray, portion of the apex with evidence of a diffuse ground-glass pattern, peripheral predominance, and bilateral pleural effusion.

**Figure 2:** Patient's chest X-ray, 1/3 average with evidence of a diffuse ground-glass pattern, also with peripheral predominance and bilateral pleural effusion.

An orotracheal intubation was performed, placed under controlled blood pressure, positive end expiratory pressure (PEEP) 9, respiratory rate of 20 irpm, tidal volume of 300, fraction of inspired oxygen (FiO) of 30%, with a PO_2_/FiO_2_ relation of 388. Nasopharyngeal and oral swabs were also collected, and a PCR was performed to detect COVID-19 and H1N1. Later, the patient was referred for a C-section and urgent sterilization. The surgery was performed under general anesthesia.

After the operation, the patient was sent to the ICU, remaining sedated, considering a −5 Rass scale. She evolved with hemodynamic instability and a capillary refill time of more than 2 seconds, requiring the introduction of vasoactive substances (norepinephrine and dobutamine). The following prescription was then begun: olsetamivir, 75 mg 12/12 h; azithromycin, 500 mg/day; hydroxychloroquine, 400 mg 12/12 h; piperacillin-tazobactam, 4.5 g 6/6 h; furosemide, 2 ampoules 8/8 h; carbegoline in a single dose to inhibit lactation; and prophylaxis for pulmonary thromboembolism (PTE) with enoxaparin, 40 mg/day.

After the birth, a chest X-ray was performed, which showed the presence of consolidation, standard air bronchogram in a ground-glass pattern in the bilateral superior lobes, with discrete bilateral pleural effusion.

On April 1, 2020, the patient showed clinical improvement, hemodynamic stability, with the possibility to suspend the vasoactive drugs, still under mechanical ventilation, but now on pressure support, presenting an increase in the PO_2_/FiO_2_ relation of 644. Sedation was paused in the morning.

On April 2, 2020, the patient was extubated, and a catheter for low-flow oxygen was implemented. The daytime laboratory showed: hemoglobin, 8.4; hematocrit, 255; leukocytes, 10.190, segmented, 68%; platelets, 137.000; arterial-blood gas, 7.43; PCO_2_, 35; PO_2_, 119; base excess, 1; bicarbonate, 24; SatO_2_, 99%; lactate, 1.0.

On the same day, the PCR exam for COVID-19 was positive. On April 2, 2020, the patient was transferred to the Fundação Santa Casa de Misericórdia do Pará, a reference service for the handling of COVID-19 patients. There, the patient underwent treatment with olsetamivir, 150 mg/day, chloroquine diphosphate, 450 mg/day; azithromycin, 500 mg/day; and piperacillin-tazobactam, 18 g/day. On April 4, 2020, she evolved to a supraventricular tachyarrhythmia, and on April 7^th^, she presented acute respiratory failure, again requiring invasive mechanical ventilation, in addition to sedation and curarization.

The patient was placed in pronation on April 9, 2020, for 16 hours, but the ventilatory asynchrony and a low PO_2_/FiO_2_ relation were maintained, even after high PEEP and FiO_2_ levels. On April 11, 2020, the patient was kept on ventilatory asynchrony, SatO_2_ of 50%, and a low PO_2_/FiO_2_ relation. She then evolved to a cardiac arrest with asystole, with an unsuccessful attempt to revive the patient, who died at 10:10am.

The newborn was born in bradycardia and fetal distress, requiring resuscitation; Apgar 2/6/7, clear and smooth amniotic liquid, with a birth weight of 2.750 kg, birth length of 50 cm, cephalic, thoracic, and abdominal perimeter de 34, 32, and 31 cm, respectively. He was sent to the neonatal ICU upon birth and remained under mechanical ventilation from March 30^th^ to April 8^th^, 2020. He continued to be hospitalized until April 21, 2020, when, fully recovered, he received hospital discharge. A COVID-19 exam was performed in the state of Rio Grande do Norte, Brazil, on April 21, 2020, after confirmation from the maternal diagnosis by PCR, but the result was negative.

## Conclusions

Few studies exist in the literature about the impact of the new Coronavirus in pregnant women, given that the SARS and MERTS pandemics, which occurred before the COVID-19 pandemic, were geographically limited. This was the first case of a pregnant woman with SARS COVID-19 in the northern region of the state of Pará, a fact that was aggravated by gestational cardiopathy associated with an infectious clinical condition.

The lack of data on infections caused by COVID-19 in cardiopathic pregnant women makes it imperative to study and understand how this disease behaves in this patient group and what the possible consequences both for the mother and the newborn are.
